# X-Ray Irradiation Improved WSe_2_ Optical–Electrical Synapse for Handwritten Digit Recognition

**DOI:** 10.3390/nano15181408

**Published:** 2025-09-12

**Authors:** Chuanwen Chen, Qi Sun, Yaxian Lu, Ping Chen

**Affiliations:** Center on Nano-Energy Research, Guangxi Key Laboratory for Relativistic Astrophysics, School of Physical Science and Technology, Guangxi University, Nanning 530004, China; chuanwenchen@st.gxu.edu.cn (C.C.); qisun@st.gxu.edu.cn (Q.S.); yaxianlu@st.gxu.edu.cn (Y.L.)

**Keywords:** X-ray irradiation, WSe_2_ synaptic device, synaptic plasticity, neuromorphic computing

## Abstract

Two-dimensional (2D) materials are promising candidates for neuromorphic computing owing to their atomically thin structure and tunable optoelectronic properties. However, achieving controllable synaptic behavior via defect engineering remains challenging. In this work, we introduce X-ray irradiation as a facile strategy to modulate defect states and enhance synaptic plasticity in WSe_2_-based optoelectronic synapses. The introduction of selenium vacancies via irradiation significantly improved both electrical and optical responses. Under electrical stimulation, short-term potentiation (STP) exhibited enhanced excitatory postsynaptic current (EPSC) retention exceeding 10%, measured 20 s after the stimulation peak. In addition, the nonlinearity of long-term potentiation (LTP) and long-term depression (LTD) was reduced, and the signal decay time was extended. Under optical stimulation, STP showed more than 4% improvement in EPSC retention at 16 s with similar relaxation enhancement. These effects are attributed to irradiation-induced defect states that facilitate charge carrier trapping and extend signal persistence. Moreover, the reduced nonlinearity in synaptic weight modulation improved the recognition accuracy of handwritten digits in a CrossSim-simulated MNIST task, increasing from 88.5% to 93.75%. This study demonstrates that X-ray irradiation is an effective method for modulating synaptic weights in 2D materials, offering a universal strategy for defect engineering in neuromorphic device applications.

## 1. Introduction

Over recent decades, computing systems based on von Neumann architecture—featuring physical separation of processing and memory units—have supported massive data processing and fueled the information age. However, in the era of big data and artificial intelligence, inherent limitations of this architecture have become pronounced: frequent data shuttling results in high energy consumption and low efficiency, limiting the rate of information exchange [1,2,3]. Biological neural systems, with their integrated sensing–computing–memory parallel processing, demonstrate remarkable advantages in energy efficiency and adaptive learning [4,5]. Inspired by this, artificial synaptic electronic and optoelectronic devices enabling neuromorphic computing have been extensively studied [6,7,8,9,10,11].

Among candidate materials, 2D materials such as WSe_2_ are ideal for constructing artificial synapses due to their unique optoelectronic properties including fast response, high responsivity, and atomic-scale thickness [12,13,14,15,16,17]. Nevertheless, the intrinsic properties of 2D materials struggle to fully emulate the complex dynamic plasticity of biological synapses, which relies on precise spatiotemporal regulation of ion gradients and neurotransmitter concentrations [18]. To achieve biomimetic dynamic responses in solid-state devices, defect engineering emerges as a core approach for tuning material properties [19,20], which is widely employed to optimize dynamic responses and energy efficiency in synaptic devices [21,22]. Several methods have been explored for defect introduction, including chemical doping/gas treatment, plasma treatment, ion/electron beam irradiation, van der Waals integration/interface defect engineering, and so on. Chemical doping/gas treatment modifies electronic structures via gas molecule adsorption or high-temperature annealing [23,24,25,26,27], which requires high temperatures/vacuum and suffers from uniformity issues. Plasma treatment involves bombarding surfaces with O_2_, Ar, or SF_6_ to selectively remove atoms or introduce functional groups [28], facing the disadvantage of surface over-etching. Ion/electron beam irradiation involves inducing atomic displacement or defect reconstruction via high-energy particle beams [29,30,31]. Van der Waals integration/interface defect engineering involves creating controlled defect states at incoherent heterojunction interfaces [32,33]; however, complex processes for interface defect engineering hinder widespread application. In contrast, X-ray irradiation offers a non-contact, high-penetration defect-modulation technique to controllably introduce selenium vacancies or interstitial defects in WSe_2_ [34,35,36,37]. For example, Choi et al. generate Te vacancies preferentially in MoTe_2_ via photoreduction (TeO_2_ → Te^0^) by micro-focused X-rays, in which the severe photon-induced reduction in the p-dopant TeO_2_ in MoTe_2_ causes the upward shift in the Fermi level (EF) due to electron injection [38]. Kolhe et al. reported a reduction in the optical bandgap from 1.60 eV to 1.14 eV and a significant increase in current upon irradiating WSe_2_ films with γ-rays, which was attributed to defect formation [39]. The changed bandgap and enhanced currents suggest the potential of X-ray irradiation for modulating synaptic plasticity, which remains unexplored.

Herein, synaptic performance of WSe_2_ devices was modulated via X-ray irradiation successfully, demonstrating X-ray irradiation is an effective strategy to regulate synaptic plasticity. Enhanced short-term potentiation (STP) and long-term potentiation (LTP) are achieved after irradiating X-ray for 2 min, which is attributed to regulated carrier concentration/transport regulated by defect states through gate and optical pulses. Excitatory postsynaptic current (EPSC) was enhanced by more than 10% and more than 4% after X-ray irradiation when triggered with electrical and optical pulses, respectively. In addition, under electrical pulses, the nonlinearity indices of LTP and long-term depression (LTD) decreased from 1.5695 to 0.609 and from 3.6038 to 3.1523, respectively, indicating enhanced performance in neuromorphic computing. Handwritten digit recognition demonstrated improved accuracy for 28 × 28 pixels images, rising from 88.5% to 93.75%. Our research reveals the potential of X-ray irradiation for defect modulation and synaptic enhancement in WSe_2_, offering a low-damage, high-compatibility paradigm for neuromorphic engineering.

## 2. Materials and Methods

### 2.1. WSe_2_ Synthesis

Two-dimensional layer WSe_2_ were fabricated via physical vapor deposition (PVD). High-purity WSe_2_ powder (Alfa Aesar, 99.9%, Shanghai, China) was placed in a quartz boat at the center of a tube furnace. And the 285 nm SiO_2_/Si substrate (Lijing Electronics Co., Ltd., Shenzhen, China) were positioned 18 cm downstream. Then, the furnace was heated to 1190 °C at 10 °C/min and held for 5 min for deposition under 27 sccm Ar flow. Finally, furnace was cooled naturally to room temperature.

### 2.2. Device Fabrication

SiO_2_/Si substrates with PVD-grown WSe_2_ were spin-coated with photoresist (MICROPOSIT S1805 G2, Rohm and Haas Electronic Materials K.K., Tokyo, Japan) at a rate of 3000 rpm for 45 s, followed by baking at 110 °C for 2 min. The electrode patterns were then defined using laser direct writing (Microwriter ML3, Durham Magneto Optics Ltd., Oxford, UK), and the exposed photoresist was developed using MICROPOSIT MF-319 (Rohm and Haas Electronic Materials K.K., Tokyo, Japan). Finally, silver electrodes were deposited by e-beam evaporation (PVD-75, Kurt J. Lesker Company, Jefferson Hills, PA, USA), and the remaining photoresist was removed with acetone.

### 2.3. Morphology and Optical Characterization

Morphology characterization of prepared WSe_2_ was characterized by optical microscopy (Olympus BX43F, Tokyo, Japan) and AFM (Bruker Dimension Icon, Shah Alam, Malaysia). Raman spectrum was performed on a Raman spectroscopy (Horiba iHR550, Loos, France, 532 nm laser). Electrical measurements were conducted by a semiconductor parameter analyzer (Keithley 4200A-SCS, Solon, OH, USA). X-ray irradiation was carried out using an FLS-XrayV system (Beijing Zolix Instruments Co., Ltd., Beijing, China) operated at 10 kV and 200 mA. All electrical measurements were performed at room temperature.

### 2.4. Neural Network Simulation

To evaluate the pattern recognition capability of the proposed synaptic device, deep neural network (DNN) simulations were performed in Python 3.6.13 using CrossSim (version 0.2.0; Sandia National Laboratories, Albuquerque, NM, USA), an open-source crossbar-array simulator. The simulation was based on the 28 × 28 MNIST handwritten digit dataset, where each pixel was used as an input neuron. The experimental device conductance states were extracted and formatted into a non-ideal conductance lookup table to accurately reflect device behavior during network training. A standard fully connected architecture was used, consisting of three layers: an input layer with 784 neurons, a hidden layer with 300 neurons, and an output layer with 10 neurons corresponding to digits 0–9. The input and hidden layers adopted the sigmoid activation function, while the output layer used SoftMax for classification. The network was trained for 20 epochs. The simulations incorporated nonlinearity and asymmetry in the conductance update to match the experimental synaptic response.

## 3. Results and Discussion

**Figure 1:**
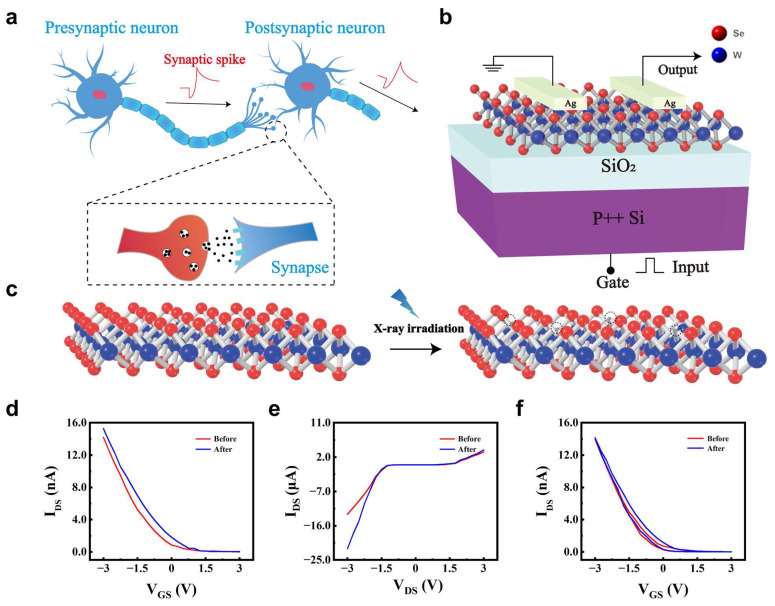
Structure and electrical characterization of WSe_2_ synaptic transistor. (**a**) Schematic of biological synapse Blue denotes the presynaptic and postsynaptic neurons. The red trace indicates the synaptic spike. The black arrow shows the direction of neurotransmitter release across the synaptic cleft. Black dots represent neurotransmitter molecules. (**b**) Device architecture of WSe_2_ FET. Red spheres denote Se and blue spheres denote W. (**c**) Defect evolution in WSe_2_ atomic layers before/after X-ray irradiation. Virtual hollow circles mark Se vacancies generated by X-ray irradiation. (**d**) Transfer curves of WSe_2_ FET before/after X-ray irradiation. (**e**) Output curves of WSe_2_ FET before/after X-ray irradiation. (**f**) Transfer hysteresis of WSe_2_ FET before/after X-ray irradiation.

Synapse, mediating information transfer between neurons, is the basic component in biological neural networks, where action potentials trigger voltage-gated Ca^2+^ channels at presynaptic terminals, releasing neurotransmitters that diffuse across the synaptic cleft to activate receptors on the postsynaptic membrane (Figure 1a). The modifiable connection strength between pre- and post-synaptic neurons defines synaptic plasticity [40]. Thus, we chose WSe_2_ as channel to achieve synaptic performance and modulate synaptic plasticity by X-ray irradiation. A three-terminal transistor structure was employed [41] (Figure 1b), where the gate electrode serves as the presynaptic terminal to regulate Ca^2+^, and the WSe_2_ channel functions as the postsynaptic terminal. WSe_2_ layers were prepared by physical vapor deposition (PVD) [42]. And the thickness of prepared WSe_2_ was characterized by atomic force microscopy (AFM), presenting 1.65 nm and indicating bilayer structure of WSe_2_ (Figure S1a). The crystal structure of prepared WSe_2_ was conducted by Raman spectroscopy (Figure S1b), showing peaks at 251 cm^−1^ (E_2g_^1^), 259 cm^−1^ (A_1g_), and 310 cm^−1^ (B_2g_^1^), which are ascribed to the intralayer vibration of E_2g_^1^, A_1g_, and interlayer interactions B_2g_^1^, respectively [43,44]. After X-ray irradiation, the Raman intensities of the E_2g_^1^, A_1g_, and B_2g_^1^ modes decreased while their peak positions remain unchanged, accompanied by a slight broadening of the full width at half maximum (FWHM). The Raman intensities of the E_2g_^1^, A_1g_, and B_2g_^1^ modes reduced continuously with the increase in irradiation time from 0 to 10 min. The reduced intensities and broadening of FWHM indicates added defect concentration in WSe_2_ induced by X-ray irradiation, such as Se vacancies, for it is the lowest formation energy [45]. The added Se vacancies enhanced defect scattering, leading to FWHM broadening and a reduced resonance Raman cross-section [46,47]. Field effect transistor (FET) was fabricated by depositing silver on WSe_2_ via laser direct writing and e-beam evaporation (Figure S1c).

Then, transfer characteristics and hysteresis curves were performed on WSe_2_ FET (Figure 1d) to evaluate the potential synaptic simulation. Before X-ray irradiation, drain current (I_DS_) increases in transfer curve as gate voltage (V_G_) sweeps from positive 3 V to negative −3 V, indicating p-type channel behavior. After X-ray irradiation, the transfer curve exhibits a significant positive shift [48], indicating a smaller V_G_-induced equivalent hole accumulation at negative V_G_ and the enhanced hole modulation efficiency by X-ray irradiation. Because X-ray irradiation induced Se vacancies in WSe_2_ (Figure 1c) [38], which presented positive electrical properties and trapped electrons in WSe_2_ channels, resulting in a higher hole concentration and currents [49]. Before X-ray irradiation, the output characteristics of the WSe_2_-based device exhibited pronounced nonlinearity at zero gate bias (Figure 1e), indicating the presence of Schottky contacts between the electrodes and the WSe_2_ channel. Additionally, the output curves measured under negative gate voltages were significantly higher than those under positive gate voltages. This asymmetry is attributed to electron trapping under positive gate bias, which reduces the hole concentration in the channel. In contrast, applying a negative gate voltage facilitates the release of trapped electrons, thereby increasing the hole concentration (Figure S2).

After X-ray irradiation, the output current was further enhanced, consistent with carrier concentration modulation induced by irradiation-generated defects. Specifically, when a V_G_ of 3 V was applied, the I_DS_ increased from 3.37 μA to 3.89 μA. At −3 V, the absolute value of the I_DS_ increased from 13 μA to 22 μA, confirming the role of X-ray-induced defects in modulating charge transport.

Before X-ray irradiation, hysteresis is observed in the transfer characteristics of the WSe_2_-based artificial synapse (Figure 1f) when the V_G_ is swept from +3 V to −3 V and then back to +3 V, demonstrating synaptic-like behavior [50]. Following irradiation, the hysteresis window becomes significantly wider (Figure 1f). To further clarify the origin of the wider hysteresis, transfer hysteresis curves were measured with three gate voltage steps at 0.1, 0.5, and 1.0 V sweeping from +4 V to −4 V (Figure S3a,b). Transfer hysteresis did not change obviously with increasing the sweeping rate (Figure S3c), suggesting interface defects are not the main reason for the wider transfer hysteresis for X-ray-irradiated WSe_2_ field effect transistors [51]. The threshold voltage shifts from −1.1 V to −0.3 V with increasing X-ray irradiation, indicating defects within WSe_2_ response to the wider transfer hysteresis (Figure S3d,e). Because Se vacancies in WSe_2_ tend to induce positive shifts in threshold voltage and Raman peak broadening due to charge carrier trapping [52], which is consistent with reduced Raman intensities in Figure 1b [46]. Thus, the electrons trapped in selenium vacancies exhibit a slower release and recovery process during the reverse gate sweep, contributing to the enhanced hysteresis effect.

**Figure 2:**
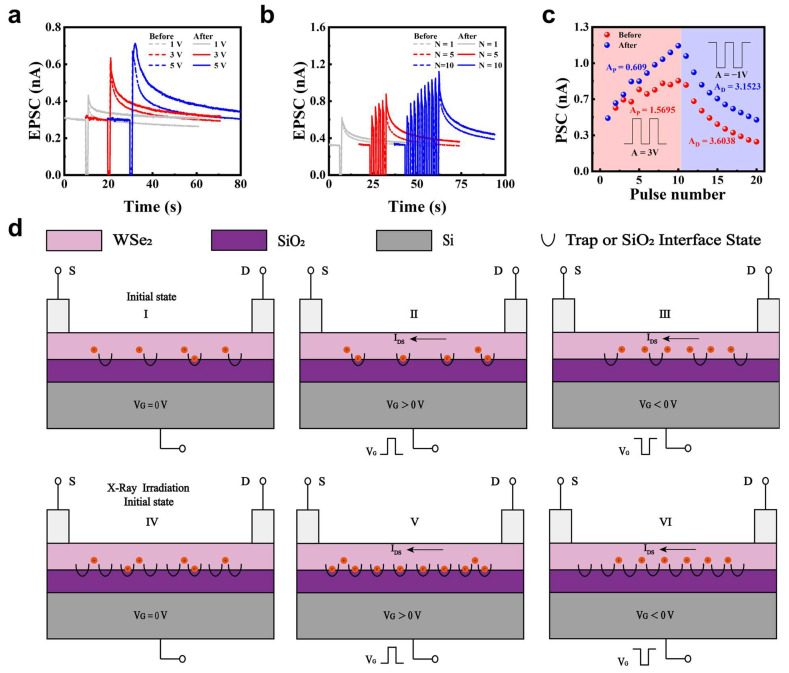
Electrically modulated synaptic plasticity and mechanism in WSe_2_ artificial synapses. (**a**) EPSC under varied pulse amplitudes before and after irradiation (W = 1 s, Δt = 1 s). (**b**) Transition from STP to LTP under different numbers of electrical pulses (3 V, W = 1 s, Δt = 1 s) before and after X-ray irradiation. (**c**) LTP and LTD triggered by 10 positive gate pulses (3 V, W = 1.5 s, Δt = 0.5 s) and 10 negative pulses (−1 V, W = 1 s, Δt = 1 s) before and after irradiation. (**d**) Mechanism schematic of defects trapped EPSC.

To demonstrate synaptic weight updates, we first simulated EPSC responses triggered by single gate voltage pulses with varying amplitudes (Figure 2a) and pulse widths (W) (Figure S4d). When a positive gate voltage pulse was applied, electrons were injected into the WSe_2_ channel, resulting in a rapid decrease in current. A portion of these electrons were trapped by defects in the WSe_2_ layer or at the WSe_2_/SiO_2_ interface [53,54]. After the gate voltage pulse was removed, electron injection ceased, and the trapped electrons induced an increase in hole concentration, thereby enhancing the postsynaptic current (PSC) (Figure 2d, State II). This led to a rapid rise in PSC. Subsequently, the PSC gradually decreased and returned to its initial state due to the slow release of electrons from the defect states. This dynamic behavior of PSC manifested as EPSC characteristics. The EPSC amplitude increased with higher positive gate voltages, attributed to the increased number of trapped electrons. X-ray irradiation was subsequently applied to the WSe_2_-based artificial synapse, leading to a further enhancement of EPSC peaks. The current increased from 0.37 nA to 0.43 nA, from 0.60 nA to 0.635 nA, and from 0.67 nA to 0.71 nA, which is attributed to the formation of additional defect sites in the WSe_2_ channel (Figure 2d, State V). Correspondingly, EPSC retention measured 20 s after STP was triggered and improved by 15.37%, 10.92%, and 15.68% under gate voltage amplitudes of 1 V, 3 V, and 5 V, respectively (Figure S4a–c), demonstrating the effective modulation of synaptic weight by X-ray irradiation.

In addition, the transition from STP to LTP was achieved by applying 1, 5, or 10 gate pulses (3 V, W = 1 s, Δt = 1 s) (Figure 2b), which is attributed to the incomplete release of electrons trapped during the previous stimulation [14]. Following X-ray irradiation, both EPSC peak values and relaxation times were further enhanced (Figure 2b), confirming a cumulative process leading to LTP. A similar STP-to-LTP transition was also observed under the same pulsing conditions (Figure S4e).

Furthermore, memory formation and decay processes were simulated and modulated by X-ray irradiation. Before irradiation, LTP, corresponding to a learning process, was induced by applying 10 positive voltage pulses (3 V, W = 1.5 s, Δt = 0.5 s), as illustrated in Figure 2c. Subsequently, 10 negative pulses (−1 V, W = 1 s, Δt = 1 s) triggered LTD, representing a forgetting process. Meanwhile, both smaller voltages and reduced pulse width evoke distinct changes in PSC, demonstrating tunable plasticity (Figure S4f,g). After X-ray irradiation, the PSC for LTP and LTD increased obviously. The reason is that higher defect density traps more electrons under identical positive pulses, elevating hole concentration and enhancing EPSC peaks/relaxation times (Figure 2d, State VI). Meanwhile, a longer term of complete release existed for more trapped electrons in defects under negative pulses. The modulated PSC for LTP and LTD indicate the regulated ability of synaptic weight by X-ray irradiation.

To quantify the linearity of weight updates during LTP and LTD, we implemented an LTP/LTD nonlinear model using the DNN NeuroSim V2.0 framework [55] to fit the device parameters [56]. The conductance changes (*G_LTP_* and *G_LTD_*) are modeled as functions of pulse number (*P*) as follows:

(1) $$G_{LTP}=B\left(1-e^{\left(-\frac{P}{A}\right)}\right)+G_{\min}$$

(2) $$G_{LTD}=-B\left(1-e^{\left(\frac{P-P_{\max}}{A}\right)}\right)+G_{\max}$$

(3) $$B=\left(G_{\max}-G_{\min}\right)/\left(1-e^{\frac{-P_{\max}}{A}}\right)$$

*G_LTP_* and *G_LTD_* are the conductance for LTP and LTD, respectively. *G*_max_, *G*_min_, and *P*_max_ are directly extracted from the experimental data, which represents the maximum conductance, minimum conductance, and the maximum pulse number required to switch the device between the minimum and maximum conductance states. *A* is the parameter that controls the nonlinear behavior of weight update. B is simply a function of *A* that fits the functions within the range of *G*_max_, *G*_min_, and *P*_max_. LTP nonlinearity decreased from 1.5695 to 0.609, and LTD nonlinearity from 3.6038 to 3.1523 after irradiation (Figure 2c).

**Figure 3:**
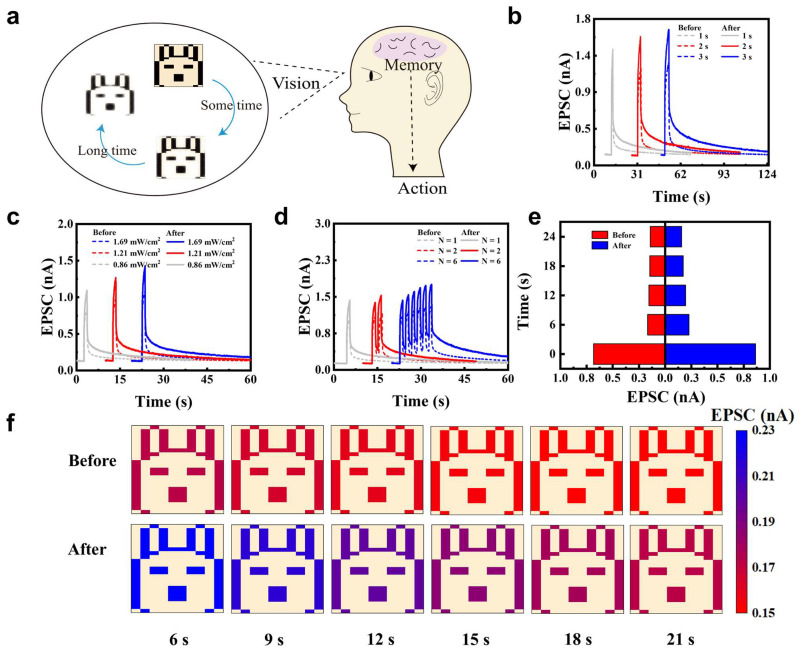
Optically modulated synaptic plasticity in WSe_2_ artificial synapses for biomimetic vision: STP, LTP, and forgetting. (**a**) Human visual perception. (**b**) STP under varied pulse widths at 1.69 mW/cm^2^ before and after irradiation. (**c**) STP characteristics under various optical power densities before and after X-ray irradiation (W = 1 s, Δt = 1 s). (**d**) Transition from STP to LTP achieved by increasing the number of optical pulses before and after X-ray irradiation (W = 1 s, Δt = 1 s). (**e**) EPSC at low power optical pulse (0.48 mW/cm^2^, W = 1 s, Δt = 1 s) before and after irradiation. (**f**) Simulated forgetting of an observed image over time before and after irradiation.

Considering the unique band structure and light–matter interactions of layered WSe_2_ [57], optical synaptic functions modulated by X-ray irradiation were investigated. Optical synapses play a crucial role in the human visual system (Figure 3a) [58]. In the human visual system, visual signals are initially captured by retinal photoreceptors and subsequently undergo preprocessing through intraretinal synaptic circuits. This process—comprising dynamic range compression, spatiotemporal filtering, and feature extraction—effectively reduces redundant information before it reaches the brain. Inspired by this biological mechanism, optical synapses aim to emulate such synaptic preprocessing functions by directly responding to light stimuli and modulating signal strength accordingly. Through this functionality, artificial optical synapses hold great potential for enabling neuromorphic visual systems that integrate sensing, processing, and memory in a compact platform [58,59,60]. To validate the optoelectronic synaptic properties, we tested the device using optical pulses with a wavelength of 532 nm and a power density of 1.69 mW/cm^2^ (Figure 3b). Upon light illumination, the EPSC increased rapidly, followed by a fast initial decay and a slower secondary decay phase. During this process, WSe_2_ absorbed photons with a wavelength of 532 nm, exciting electrons from the valence band to the conduction band and generating abundant photoinduced carriers, which resulted in a significant increase in current. Simultaneously, Se vacancies preferentially captured photogenerated electrons, promoting hole-dominated conduction and causing the rapid rise in EPSC. After cessation of illumination, untrapped carriers recombined rapidly, while trapped electrons were released slowly, leading to a gradual decrease in hole concentration in the channel, corresponding to the slow decay phase of the forgetting process. EPSC increased with widening pulse widths, attributed to added photocarriers generated by the widened pulse widths. Then, EPSC increased significantly after X-ray irradiation from 1.09 nA, 1.2 nA, 1.28 nA to 1.43 nA, 1.58 nA, 1.67 nA, respectively, with pulse widths at 1 s, 2 s, and 3 s. This phenomenon originated from the increased defect density in WSe_2_ induced by X-ray irradiation, leading to more electrons being trapped during illumination and a higher hole concentration, thus a larger EPSC. Furthermore, after irradiation, both the peak EPSC and the relaxation time increased under each pulse width condition. Additionally, the EPSCs were improved by 4.56%, 5.12%, and 7.69% in retention at 16 s for pulse widths of 1 s, 2 s, and 3 s, respectively (Figure S5a–c). The same regulated phenomena were shown with different excited powers and the number of optical pulses (Figure 3c,d). To evaluate the regulated EPSC by X-ray irradiation qualitatively, the EPSCs before and after irradiation excited by 532 nm with low power optical pulse (0.48 mW/cm^2^, W = 1 s, Δt = 1 s) were compared (Figure 3e,f). After increasing the optical pulse, EPSC peak after irradiation is significantly larger than that without irradiation. The current decays kept higher than those without irradiation even at 21 s. Figure 3f demonstrates the device’s recognition and slow forgetting process for a rabbit contour light signal. EPSCs were mapped to a rabbit image with the current at 6 s, 9 s, 12 s, 15 s, 18 s, and 21 s, visually illustrating the forgetting process over time. After irradiation, the current exhibits a higher EPSC peak and longer forgetting time, indicating stronger image information retention capability. The enhanced EPSC and relaxation time modulated by X-ray irradiation demonstrate that defect engineering simultaneously optimizes synaptic behavior in both electrical and optical modes, suggesting signal conversion and significant potential in sensing applications.

**Figure 4:**
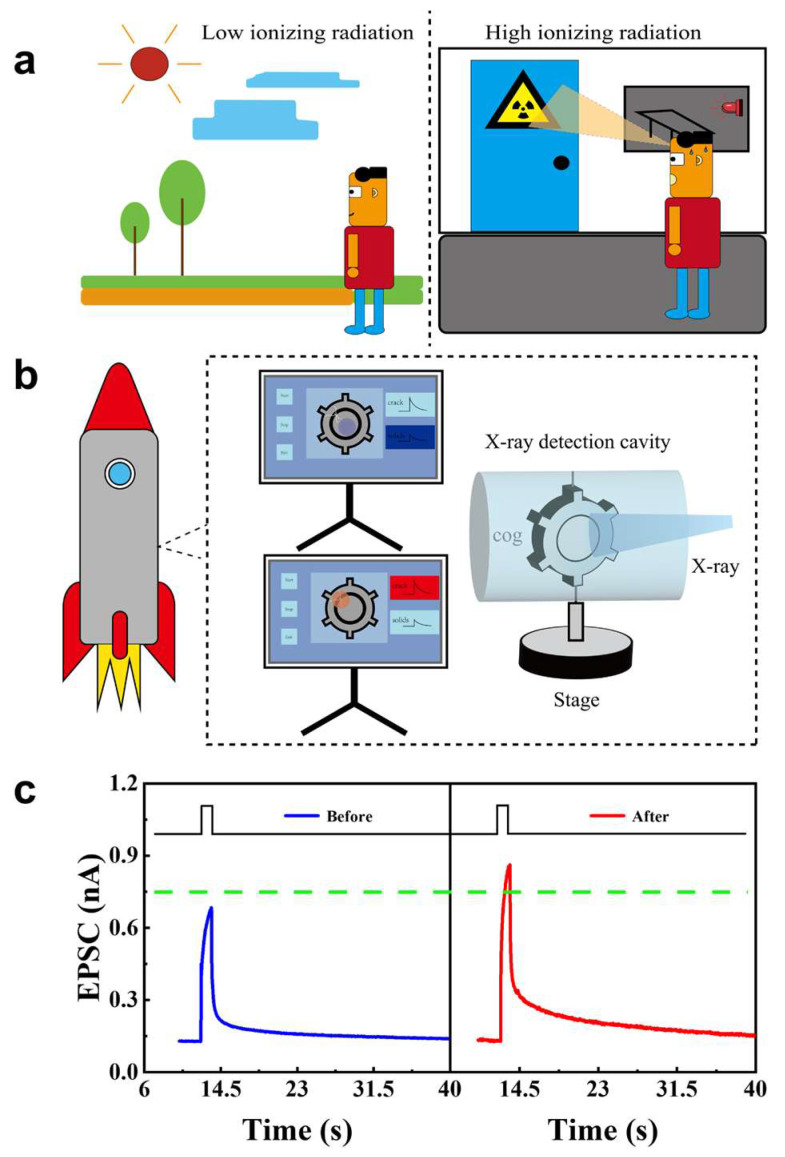
Proposed sensing applications of WSe_2_ synapse. (**a**) Schematic of X-ray monitoring in surroundings. (**b**) Schematic of structural crack detection of the gear. Blue denotes the solid region with no cracks. Red denotes cracks or defective regions. (**c**) EPSCs of WSe_2_ synapse before and after X-ray irradiation, where green line represents the set threshold current. The green dashed line marks the threshold current used for classification.

Environmental X-ray radiation detection and non-destructive structural cracks monitoring were proposed based on the reaction of the WSe_2_ synapse irradiated by X-ray (Figure 4). Notably, prolonged/high-dose X-ray exposure is hazardous (Figure 4a). An alarm was proposed according to the set threshold current (Figure 4c) of EPSC. The EPSC was smaller than the threshold current when excited by 532 nm with the power at 0.48 mW/cm^2^. However, a larger EPSC was presented, which exceeded the threshold current, when irradiated by X-ray, and triggered an alarm to keep away from the ionizing radiation. Additionally, non-destructive testing (NDT) of high-precision components could be explored, such as rocket engine gears (Figure 4b). If the gear is intact and crack-free, the transmitted dose of X-ray is low. Conversely, if cracks are presented in the gear, the transmitted dose will be larger and impinge on the WSe_2_ synapse. By monitoring the synapse EPSC (Figure 4c), current, which is below threshold, indicates integrity, while that above threshold signals suggests cracks in the gear.

**Figure 5:**
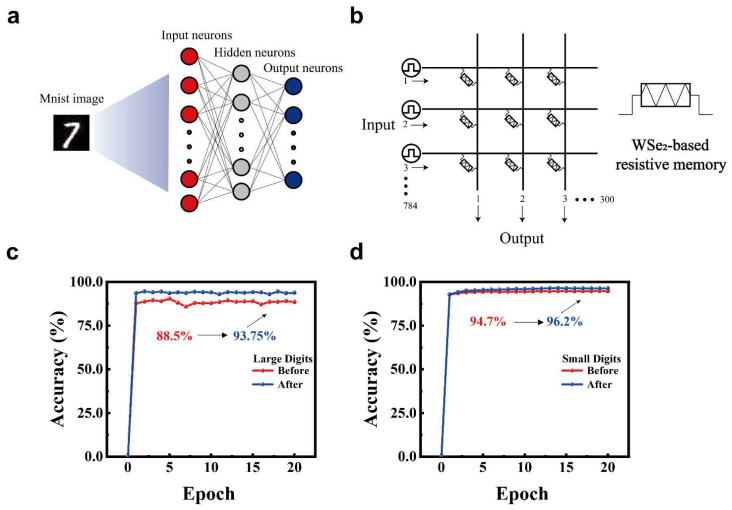
WSe_2_ artificial synapses for neural network-based handwritten digit recognition. (**a**) Schematic of the neural network architecture. (**b**) Crossbar array structure implementing synaptic weight mapping. (**c**) Recognition accuracy versus training epochs for large handwritten digit images (28 × 28 pixels). (**d**) Recognition accuracy versus training epochs for small handwritten digit images (8 × 8 pixels).

To validate the device’s applicability in neuromorphic computing, as shown in Figure 5a, we simulated a neural network using the CrossSim crossbar simulator with the MNIST dataset [61,62]. This artificial neural network (ANN) consisted of 784 input neurons, 300 hidden neurons, and 10 output neurons. A 784 × 300 crossbar array was constructed using WSe_2_ synaptic devices as memory elements (Figure 5b). The device exhibited continuously tunable conductance states under electrical pulses (Figure 2c). LTP and LTD were implemented through 10 positive gate pulses (3 V, W = 1.5 s, Δt = 0.5 s) and 10 negative gate pulses (−1 V, W = 1 s, Δt = 1 s), respectively. Figure 5c compares the recognition accuracy versus training epochs for large handwritten digit images (28 × 28 pixels) based on synaptic conductance update characteristics before and after irradiation. After irradiation, the recognition accuracy improved significantly from 88.5% to 93.75% [63]. Figure 5d demonstrates recognition accuracy improvement for small handwritten digit images (8 × 8 pixels) from 94.7% to 96.2%. Similarly, Figure S6a displays LTP triggered by eight optical pulses (1.69 mW/cm^2^, W = 1 s, Δt = 1 s) followed by LTD induced by eight electrical pulses (−1 V, W = 1 s, Δt = 1 s), with Figure S6b showing recognition accuracy examples. These simulations demonstrate the potential of X-ray-irradiated WSe_2_-based synaptic devices for constructing neural networks in neuromorphic computing applications.

## 4. Conclusions

In summary, we have demonstrated that X-ray irradiation is a powerful and controllable approach for defect engineering in WSe_2_-based artificial synapses. By introducing selenium vacancies, the synaptic performance under both electrical and optical stimulation was significantly enhanced. Notably, EPSC retention was improved, relaxation times were prolonged, and the nonlinearity of LTP/LTD processes was reduced. These enhancements directly translated into improved neuromorphic computing accuracy, with MNIST digit recognition performance increasing from 88.5% to 93.75% after irradiation. In addition, the enhanced optoelectronic response enabled practical sensing applications such as environmental X-ray monitoring and non-destructive structural diagnostics. These findings position X-ray irradiation as a general, low-damage strategy for tailoring 2D materials, paving the way for multifunctional synaptic devices that seamlessly integrate sensing, processing, and memory for future neuromorphic technologies.

## Data Availability

The original contributions presented in this study are included in the article and Supplementary Materials. Further inquiries can be directed to the corresponding author.

## Supplementary Materials

The following supporting information can be downloaded at: https://www.mdpi.com/article/10.3390/nano15181408/s1, Figure S1: Morphology and optical characterization of WSe_2_ devices. Figure S2: Output curves of WSe_2_ devices before X-ray irradiation. Figure S3: Transfer characteristics and synaptic plasticity potentiation of WSe_2_ FET under different X-ray irradiation conditions. Figure S4: Synaptic plasticity of WSe_2_ devices before and after X-ray irradiation. Figure S5: Synaptic plasticity of WSe_2_ devices simulated by optical pulses at 532 nm with the power at 1.69 mW/cm^2^. Figure S6: WSe_2_ artificial synapses for neural network-based handwritten digit recognition.
