# Supplementary material for: X-Ray Irradiation Improved WSe2 Optical–Electrical Synapse for Handwritten Digit Recognition
Source: Nanomaterials (Basel). 2025 Sep 12;15(18):1408. doi: 10.3390/nano15181408 (PMC12472380; doi:10.3390/nano15181408)
Supplement: Supplementary file 1 [file nanomaterials-15-01408-s001.zip › nanomaterials-3834469-supplementary.pdf]

Article

X-ray Irradiation Improved WSe_2 Optical–Electrical Synapse for Handwritten Digit Recognition

Chuanwen Chen, Qi Sun, Yaxian Lu and Ping Chen *

Center on Nano-Energy Research, Guangxi Key Laboratory for Relativistic Astrophysics, School of Physical Science and Technology, Guangxi University, Nanning 530004, China; chuanwenchen@st.gxu.edu.cn (C.C.); qisun@st.gxu.edu.cn (Q.S.); yaxianlu@st.gxu.edu.cn (Y.L.)

* Correspondence: chenping@gxu.edu.cn

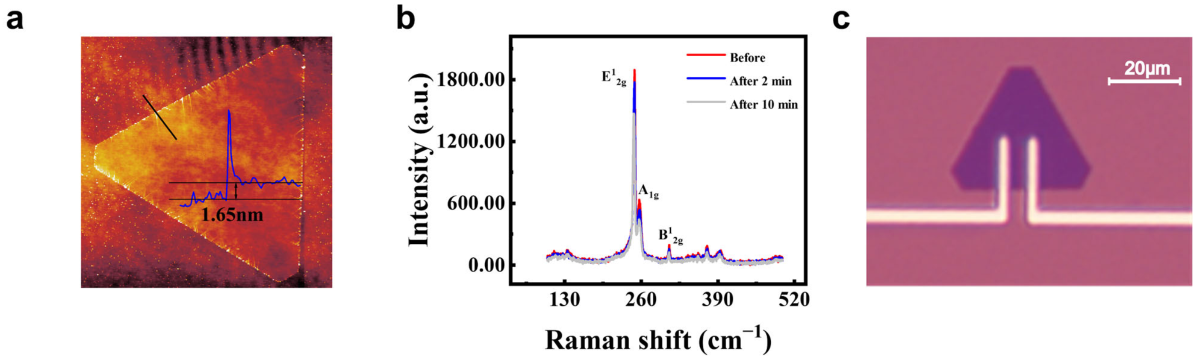

Figure S1. Morphology and optical characterization of WSe_2 devices. (a) AFM image. (b) Raman spectra of the WSe_2 before and after X-ray irradiation for 2 min and 10 min with the excitation at 532 nm. (c) Optical image of WSe_2 FET.

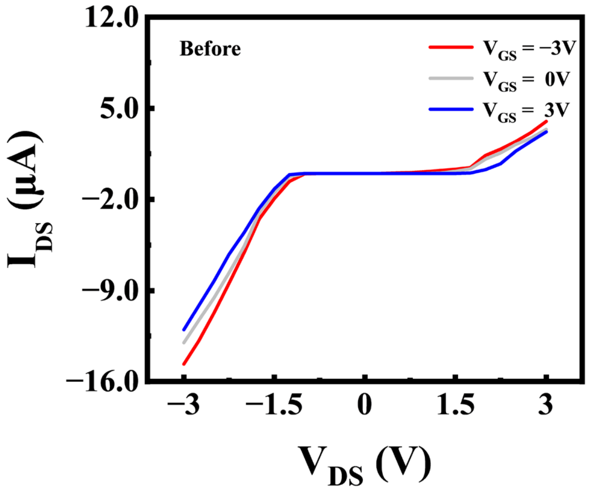

Figure S2. Output curves of WSe_2 devices before X-ray irradiation. Under different gate voltages of −3 V, 0 V, and 3 V, demonstrating typical output characteristics and ohmic contact behavior.

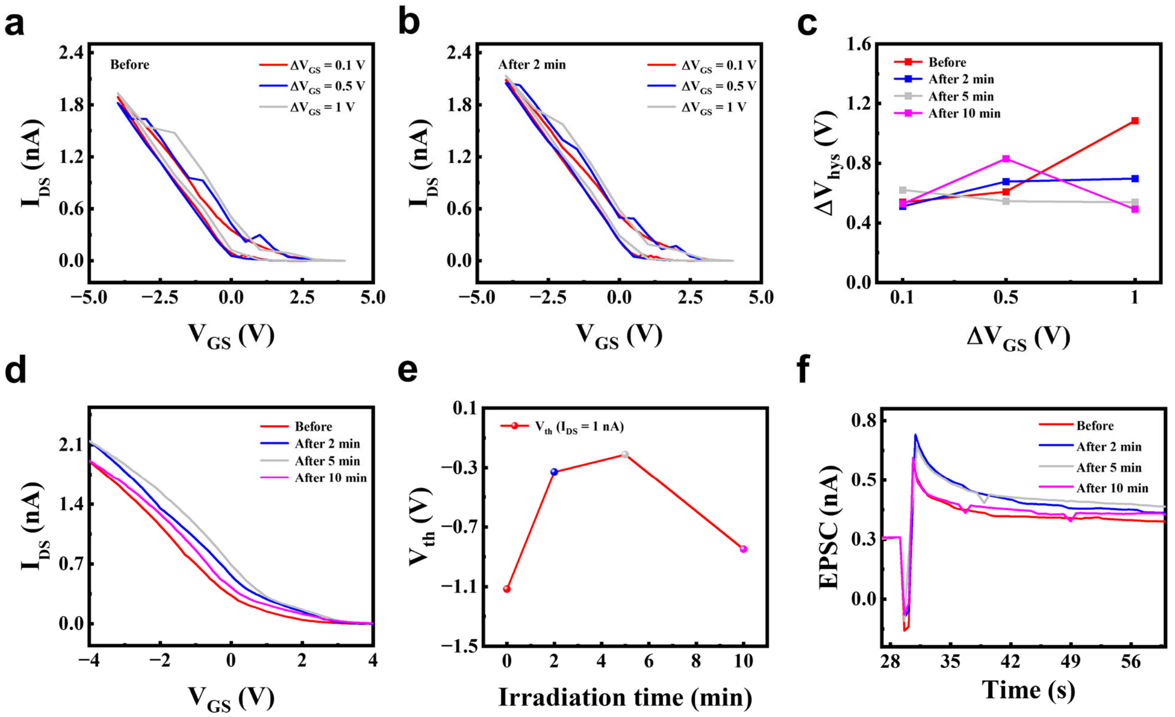

Figure S3. Transfer characteristics and synaptic plasticity potentiation of WSe_2 FET under different X-ray irradiation conditions. (a–b) Transfer hysteresis of WSe_2 FET before and after 2 min X-ray irradiation at different V_{GS} step sizes. (c) Extracted hysteresis width ΔV_{hys} as a function of V_{GS} step size. (d) Transfer curves of WSe_2 FET before and after different X-ray irradiations. (e) Changes in threshold voltage V_{th} with X-ray irradiation time. (f) EPSC under different irradiation times.

Irradiation time-dependent evolution of transfer characteristics, threshold voltage, and short-term potentiation

In order to investigate the influence of irradiation time, WSe_2-FET devices were irradiated by X-ray for 0, 2, 5, and 10 minutes. As shown in Figure S3a-b, transfer hysteresis measured at different gate voltage step sizes (0.1, 0.5, 1.0 V) exhibit hysteresis both before and after 2 min irradiation. The extracted hysteresis (ΔV_{hys}) remains nearly unchanged with increasing voltage step and increasing irradiation time (Figure S3c), suggesting that interface states are not the main contributor to the observed hysteresis. Figure S3d-e shows that the threshold voltage (V_{th}) initially shifted positively and, subsequently, negatively with increasing irradiation time. STP behavior under different irradiation times is presented in Figure S3f. These results suggest that short irradiation (2 minutes) induces moderate changes in device characteristics, while prolonged irradiation (10 minutes) causes a performance decline. This trend is likely associated with the accumulation of excessive defect states at longer irradiation durations, which may impair device stability.

To investigate the influence of irradiation time, WSe_2 FET devices were irradiated by X-ray for 0, 2, 5, and 10 minutes. As shown in Figure S3a–b, transfer hysteresis is observed both before and after 2 minutes of irradiation under various gate voltage step sizes (0.1, 0.5, and 1.0 V). The hysteresis remains nearly unchanged with increasing step size and irradiation time (Figure S3c), indicating that the contribution from defects generated at the SiO_2/WSe_2 interface is relatively minor. Figure S3d–e presents the threshold voltage with irradiation time. Threshold voltage initially shifts positively with increasing irradiation time and subsequently shifts negatively. These results suggest that short-term irradiation (2 minutes) induces moderate modulation in device characteristics, while prolonged irradiation (10 minutes) leads to performance decrease. This trend likely results from the

accumulation of excessive defect states that impair device stability. Figure S3f shows the short-term potentiation behavior under different irradiation times. The synaptic response is enhanced after short-term irradiation but decreases at 10 minutes, consistent with the threshold voltage behavior.

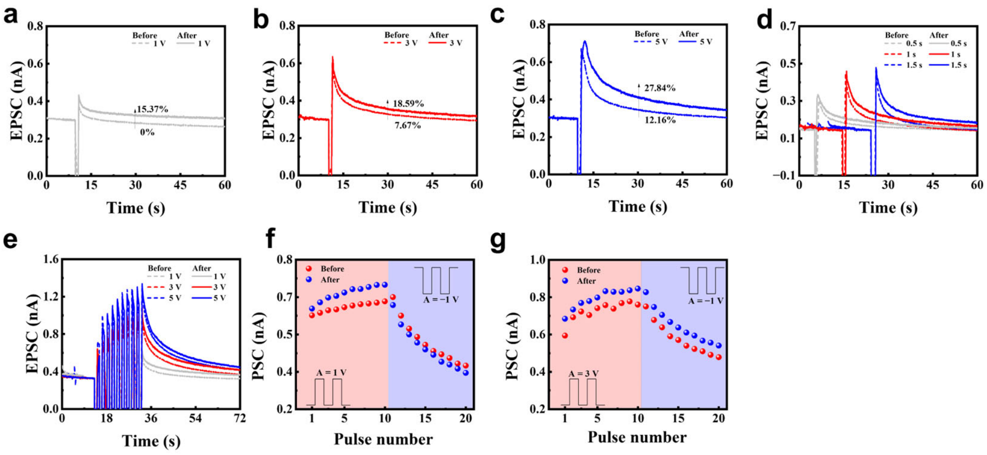

Figure S4. Synaptic plasticity of WSe_2 devices before and after X-ray irradiation. (a-c) EPSCs of WSe_2 devices triggered by a gate pulse at 1 V (a), 3 V (b), and 5 V (c). (d) EPSCs of WSe_2 devices triggered by a gate pulse with varied pulse widths. (e) Synaptic plasticity triggered by 10 positive voltage pulses under different gate voltages. (f) LTP and LTD of WSe_2 devices triggered by 10 positive gate pulses (1 V, W = 1 s, t = 1 s) and 10 negative gate pulses (−1 V, W = 1 s, t = 1 s). (g) LTP and LTD of WSe_2 devices triggered by 10 positive gate pulses (3 V, W = 0.5 s, t = 1.5 s) and 10 negative gate pulses (−1 V, W = 1 s, t = 1 s).

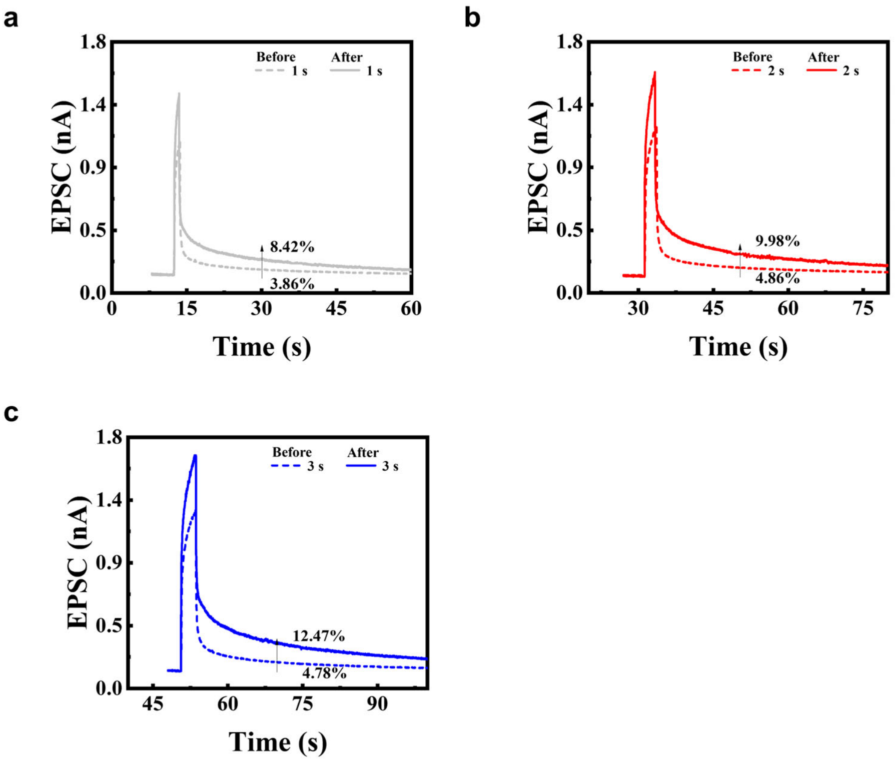

Figure S5. Synaptic plasticity of WSe_2 devices simulated by optical pulses at 532 nm with the power at 1.69 mW/cm^2: (a) EPSCs of WSe_2 devices before and after irradiation simulated by an optical pulse with width at 1 s. (b) EPSCs of WSe_2 devices before and after irradiation simulated by an optical

pulse with width at 2 s. (c) EPSCs of WSe_2 devices before and after irradiation simulated by an optical pulse with width at 3 s.

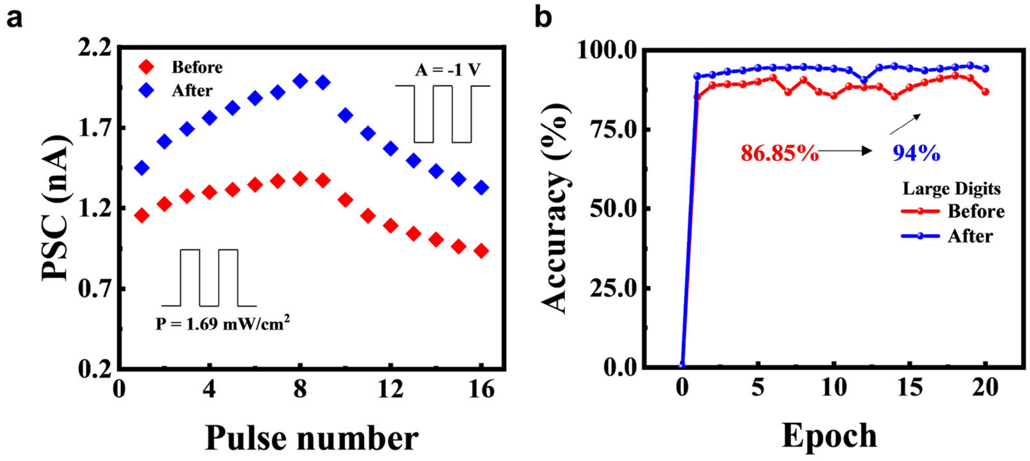

Figure S6. WSe_2 artificial synapses for neural network-based handwritten digit recognition. (a) LTP and LTD triggered by optical and electrical pulses of WSe_2 devices before and after X-ray irradiation. (b) Recognition accuracy versus training epochs for large handwritten digit images (28 × 28 pixels).
